# A virtual phantom for patient‐specific QA On A 1.5*T* MR‐linac

**DOI:** 10.1002/acm2.14264

**Published:** 2024-01-22

**Authors:** Mingshuo Ji, Zhenjiang Li, Yuan Tian, Ke Zhang, Minghui Li, Yan Chen

**Affiliations:** ^1^ Elekta, Inc. Atlanta Georgia USA; ^2^ Department of Radiation Oncology Shandong Cancer Hospital and Institute Shandong First Medical University and Shandong Academy of Medical Sciences (SDCH) Jinan China; ^3^ Department of Radiation Oncology National Cancer Center/National Clinical Research Center for Cancer Cancer Hospital Chinese Academy of, Medical Sciences and Peking Union Medical College, (CAMS) Beijing Beijing China; ^4^ Elekta Ltd. Asia Pacific Hongkong China

**Keywords:** ArcCHECK‐MR, MR‐linac, patient‐specific quality assurance, plan verification, radiotherapy

## Abstract

Create a virtual ArcCHECK‐MR phantom, customized for a 1.5*T* MR‐linac, with consideration of the different density regions within the quality assurance (QA) phantom, aiming to streamline the utilization of this specialized QA device. A virtual phantom was constructed in the treatment planning system (TPS) to replicate the ArcCHECK‐MR's composition, consisting of five distinct layers: “Outer” (representing the outer PMMA ring), “Complex” (simulating the printed circuit boards), “Detectors” (encompassing the detector area), “Inner” (signifying the inner PMMA ring) and “Insert” (representing the PMMA insert). These layers were defined based on geometric data and represented as contour points on a set of dummy CT images. Additionally, a setup platform was integrated as contoured structures. To determine the relative electron density (RED) values of the external and internal PMMA components, measurements were taken at 25 points in the insert using an ion chamber. A novel method for establishing the exit/entrance dose ratio (EEDR) for ArcCHECK‐MR was introduced. The RED of higher density region was derived by evaluating the local gamma index passing rate results with criteria of 2% dose difference and 2 mm distance‐to‐agreement. The performance of the virtual phantom was assessed for Unity 7 *FFF* beams with a 1.5*T* magnetic field. The radii of the five ring structures within the virtual phantom measured 133.0 *mm*, 110.0 *mm*, 103.4 *mm*, 100.0 *mm*, and 75.0 *mm* for the “Outer,” “Complex,” “Detectors,” “Inner” and “Insert” regions, respectively. The RED values were as follows: ArcCHECK‐MR PMMA had a RED of 1.130, “Detectors” were assumed to have a RED of 1.000, “Complex” had a RED of 1.200, and the setup QA phantom justified a RED of 1.350. Early validation results demonstrate that the 5‐layer virtual phantom, when compared to the commonly used bulk overridden phantom, offers improved capability in MR‐linac environments. This enhancement led to an increase in passing rates for the local gamma index by approximately 5 ∼ 6%, when applying the criteria of 2%, 2 *mm*. We have successfully generated a virtual representation of the distinct regions within the ArcCHECK‐MR using a TPS, addressing the challenges associated with its use in conjunction with a 1.5*T* MR‐linac. We consistently observed favorable local gamma index passing rates across two 1.5*T* MR‐linac and ArcCHECK‐MR unit combinations. This approach has the potential to minimize uncertainties in the creation of the QA phantom for ArcCHECK‐MR across various institutions.

## 1 INTRODUCTION

MR‐guided radiotherapy (MRgRT) is a cutting‐edge technology in the field of radiotherapy that delivers precise radiation through the integration of MRI and a medical linear accelerator, known as an MR‐linac. This integration enables adaptive therapy, which adjusts the treatment plan for each fraction based on the patient's anatomy at the time of treatment, leading to improved accuracy and reduced risk of side effects. MR‐linacs have seen a significant increase in clinical implementation.^1^, ^2^ However, integrating an MR‐scanner into a linac also presents new challenges for system commissioning and patient‐specific quality assurance (QA).^3^, ^4^, ^5^, ^6^, ^7^

One of the challenges in MRgRT is the electron return effect (ERE), which occurs due to the Lorentz force that causes deflection of secondary electrons, leading to changes in the delivered dose to the target and an increase in surface dose. The ERE is particularly significant in non‐homogeneous media with vastly different densities such as tissue‐air and tissue‐lung interfaces, it has been extensively studied^7^, ^8^, ^9^, ^10^, ^11^, ^12^ using single ion‐chamber and solid detectors,^13^, ^14^ as well as in specific MR‐linac QA tests.^15^ However, the impact of the ERE on measurements in patient‐specific QA devices that contain both low‐density and high‐density materials in clinical workflows has not been thoroughly examined.

The ArcCHECK‐MR (Model 1220‐MR, Sun Nuclear Corp., Melbourne, FL) is a cylindrical array detector phantom used for patient‐specific dosimetry QA in MR‐linac systems. The vendor recommends treating the device as a homogeneous phantom with a uniform density for dose calculation and adjusting the mass density or relative electron density (RED) of the phantom depending on the treatment planning system (TPS) algorithms, by using a metric of exit/entrance dose ratio (EEDR). While this approach is suitable for conventional linear accelerators (linacs), within the magnetic field of an MR‐linac, density variations in the detector array and surrounding electronics of the ArcCHECK‐MR phantom can significantly affect EEDR calculations, leading to variations in RED values for the TPS.

The goal is to characterize better the ArcCHECK‐MR in the presence of a 1.5*T* magnetic field by using a virtual phantom with varying densities, while simplify the process for individual institutes to create the phantom for patient‐specific QA.

## 2 MATERIALS AND METHODS

### 2.1 General

#### 2.1.1 Unity MR‐linac description

Unity (Elekta AB, Stockholm, Sweden) is comprised of a Philips Marlin 1.5 Tesla (T) MRI (Philips Healthcare, Best, The Netherlands) and a single‐energy 7 MV flattening filter free (7 *FFF*) standing‐wave linear accelerator, which is currently capable of delivering intensity modulated radiotherapy (IMRT) treatments. The magnetic field is perpendicular to the photon beam.^13^ Unity has a source to isocenter distance (SAD) of 143.5 cm and an inner‐bore diameter of 70.0 cm. The beam‐shaping system uses the Elekta Agility 160 leaf multileaf collimator (MLC) and a pair of V‐shape collimators. Collimator rotation is disabled. The MLC leaves move in the superior/inferior direction with respect to patient anatomy, with a leaf width of 7.2 mm at the isocenter plane. The maximum field size in the isocenter plane is 57.4 × 22.0 cm^2^. In treatment mode, the treatment table can move in and out of the bore, while its position in the vertical and lateral directions is fixed. The distance from the isocenter to the tabletop hard plate is fixed at 14.0 cm.

**FIGURE 1:**
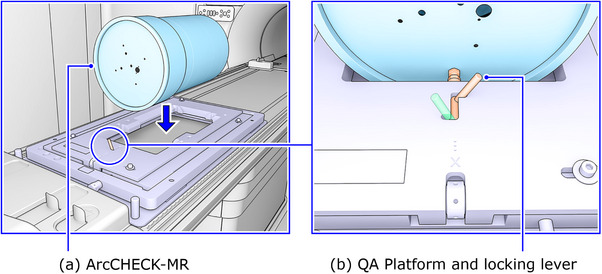
A schematic overview of the QA Platform and ArcCHECK‐MR setup. The platform consists of a top, a middle, and a bottom plate. The top and the middle plates can be adjusted separately for setting up QA devices iso‐centrically (a). There is a bracket and a locking lever to lock the ArcCHECK‐MR in position (b). (Reproduced from “Unity Instruction for Use—Clinical Mode Ver.1″, with permission from Elekta AB.).

Given the absence of an external laser alignment system in the treatment room of Unity, Elekta offers an adjustable QA Platform designed for the setup of QA devices. When properly calibrated, this platform can be securely positioned on the tabletop to accommodate QA devices like the ArcCHECK‐MR at precise locations (Figure 1).^16^ It is essential to factor in the presence of the QA Platform along the radiation paths and its potential impact on the radiation dose.

#### 2.1.2 Treatment planning parameters

Monaco Version 5.4.02 (Elekta) was used in this study. The software includes a GPU‐based Monte Carlo dose calculation algorithm (GPUMCD) that is optimized for faster calculations in online adapted planning workflows. Specifically, a set of measured cryostat characterization transmission (CCT) factors pertaining to a Unity is integrated in the GPUMCD beam model.^10^, ^17^, ^18^ For dose calculation of simple open beams, the study used a 2 × 2 × 2 mm^3^ grid size and a 0.5% statistical uncertainty per beam, while for clinical sample plans, the grid size was 2 × 2 × 2 mm^3^ and 1.0% statistical uncertainty per plan. All field sizes are reported in the IEC beam coordinate system as *FX* × *FY*.

#### 2.1.3 The ArcCHECK‐MR unit description

The ArcCHECK‐MR has 1386 SunPoint diode detectors aligned in helical geometry. The device is MR‐compatible by positioning the main data and power unit outside the 5‐gauss line with an extended cable.^3^, ^5^, ^19^, ^20^, ^21^ It can be configured with different inserts, such as a CavityPlug or MultiPlug (1220000‐1Z, or 1220000‐3Z). According to SNC, starting with SNC Patient software version 6.0, heterogeneity correction factors can be applied to the ArcCHECK‐MR measurement. To take advantage of the heterogeneity correction, the ArcCHECK‐MR should be treated as a homogeneous phantom in the TPS. Monaco users are required to make changes within the TPS, specifically by adjusting the RED assigned to the virtual ArcCHECK‐MR phantom until the TPS calculations agrees well with the EEDR derived from ArcCHECK‐MR measured data.

ArcCHECK‐MR data were collected at different times from two independent institutions, SDCH and CAMS. SDCH provided ArcCHECK‐MR 1/Unity 1, while CAMS provided ArcCHECK‐MR 2/Unity 2. Both ArcCHECK‐MR 1 and ArcCHECK‐MR 2 were equipped with individual CavityPlug inserts. Additionally, Elekta provided a MultiPlug insert for measuring multiple points’ dose in ArcCHECK‐MR 1. During setup, when an ArcCHECK‐MR was placed isocentrically with the QA platform, the source‐to‐surface distance (SSD) was set at 130.2 cm. Data acquisition was performed using SNC Patient software, version 8.3.0.2, while processing and analysis were carried out using version 8.4.0.1. Following the product specification data sheet provided by SNC, we assumed that the hardware configurations of all ArcCHECK‐MRs used in this study were identical. However, it's important to note that each unit had its dedicated array calibration factors from SNC. The SNC Patient software allowed for the analysis of passing rates using various metrics, including distance to agreement (DTA) and the *γ* index in both local and global modes, with user‐defined criteria.

#### 2.1.4 To characterize the ArcCHECK‐MR with CT images

The ArcCHECK‐MR 1, affixed to a QA platform, was subjected to imaging using a Philips BigBore CT scanner (Philips) in SDCH, followed by importing the acquired images into Monaco software. The outline of the ArcCHECK‐MR and specific components of the accompanying QA platform were delineated. Three distinct objects of the QA platform, both on the right and left sides, were contoured when they were fully visible on the CT images. If any part of the objects were missed due to limitations in the CT field of view (FOV) settings, the contours were amended based on the geometric specifications of the QA platform.

The ArcCHECK‐MR 2, affixed to an SNC frame cradle, underwent scanning using a separate Philips BigBore CT scanner equipped with dual‐energy capabilities in CAMS. Using the kV‐CT image dataset, a 3D rendering of the ArcCHECK‐MR was created by using the pyqtgraph (www.pyqtgraph.org) package within Python 3.9 (Python Software Foundation) to reveal the layout of detectors array.

A set of MV‐CT images of the ArcCHECK‐MR 2, securely affixed to a QA platform, was obtained using a Hi‐Art Tomotherapy system (Accuray, Sunnyvale, CA) at CAMS. These images were imported into Monaco to create an MV‐CT image‐based reference phantom. A MVCT‐to‐RED conversion file was generated in Monaco based on the results obtained from scanning a CIRS electron density phantom (Model 062 M, CIRS, Inc., Norfolk, VA) with known electron density inserts using the same Hi‐Art system. Subsequently, a calculation for a 10 × 10 cm^2^ beam on this phantom was performed using the GPUMCD Unity beam model in Monaco. The exported dose, along with the corresponding measured data, was analyzed using SNC Patient software. We also discuss why applying MVCT‐to‐RED conversions to ArcCHECK‐MR should be avoided.

CT pixel values were obtained from both MV and kV‐CT images of the ArcCHECK‐MR 2, along a horizontal line that runs through the center of the phantom, to characterize the CT values of the different components of the ArcCHECK‐MR.

Seven discrete density regions were identified in both the MV and kV‐CT images, ranging from the outermost to the innermost components: (1) the outer PMMA ring, (2) the outer low‐density, (3) the printed circuit boards (PCBs), (4) the detector triplets located on the inner surface of the PCBs, (5) the inner lower density regions around the detectors, (6) the inner PMMA ring, and (7) the PMMA insert. Due to the irregular shapes of the outer and inner low‐density, regions (2) and (3) were considered as one region, and regions (4) and (5) were considered as another region. Five distinct ring structures were generated as contours in the TPS, each assigned a specific name: “Outer” for the outer PMMA ring, “Complex” for the outer low‐density and PCBs, “Detectors” for the detector triplets and the adjacent low‐density areas, “Inner” for the inner PMMA ring, and “Insert” for the PMMA insert, representing either the MultiPlug or the CavityPlug inserts.

Six distinct objects characterized by varying shapes were identified on the kV‐CT images of the QA platform. These objects were further subdivided into three on each side, labeled sequentially from left to right as “LtOuter,” “LtMiddle,” “LtInner,” “RtInner,” “RtMiddle,” and “RtOuter.” However, the objects designated as “LtOuter,” “LtMiddle,” “RtMiddle,” and “RtOuter” were not fully encompassed within the CT scanner's FOV. Consequently, the missing areas were manually outlined based on the recorded geometric data of the platform.

#### 2.1.5 ArcCHECK‐MR virtual phantom

A set of 121 pseudo‐CT images was generated, each with a matrix size of 512 × 512 pixels, pixel spacing of 0.97658 mm × 0.97658 mm, slice thickness of 2 mm, and CT pixel values set to zero. These images were created using the proprietary software CarpeDICOM (Elekta). The DICOM tag “Image Position Patient” for these CT images was defined as (−249.51619, −249.51619, 0). This specific positioning ensured that the isocenter precisely coincided with the center of the ArcCHECK‐MR, aligning with the nominal geometry center of the Unity system at coordinates (0, 0, 0). This alignment facilitated the subsequent unfolding of the DICOM RT DOSE file in SNC Patient Software. The pseudo‐CT images served as the foundational DICOM CT dataset in the TPS for the virtual phantom. Contours were delineated for five distinct regions within the ArcCHECK‐MR, each with its specified radius: External (133.0 mm), Complex (110.0 mm), Detectors (103.4 mm), Inner (100.0 mm), and Insert (75.0 mm). When referencing a point within the virtual phantom, the DICOM coordinate system was employed.

We assumed minimal variation in the Relative RED of the PMMA components across different units of ArcCHECK‐MR from the same vendor. Instead of relying on measured data from ArcCHECK‐MR detectors, we conducted independent measurements to derive suitable a RED value for the PMMA components. Subsequently, the RED values for the other two regions, namely, “Detectors” and “Complex,” were deduced sequentially.

**FIGURE 2:**
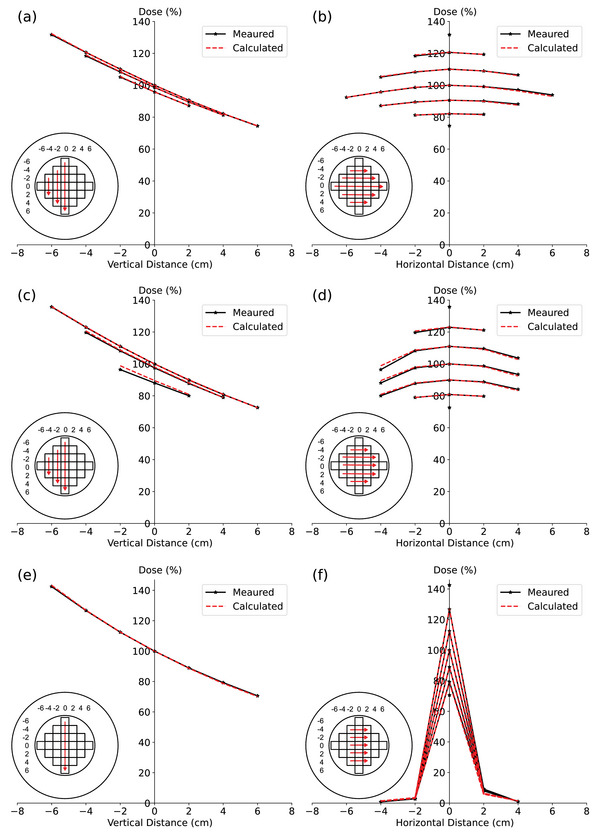
The measured and calculated data for the MultiPlug in the ArcCHECK‐MR 1 are compared to derive the RED value for the PMMA parts of the ArcCHECK‐MR. The External and Insert rings, along with the outlines of the 25 plugs, are displayed in the inserted images. Each plug is characterized by a cross‐sectional size of 2 × 2 cm^2^. Measurements were taken at the center of each plug using a SemiFlex 3D 0.07 cm^3^ detector, which was connected to a UniDos electrometer. The corresponding beams were calculated in the TPS. Data are normalized to the doses at the center of the ArcCHECK‐MR for each beam for the measured and calculated, respectively. For the beam of 40 × 22 cm^2^: (a) Depth dose curves along the columns at 0, −2 and −4 of the MultiPlug, those at 2 and 4 are not included, as they are similar to those at −2 and −4 and cannot be distinguished in the plot. (b) Cross‐plane profiles of the same beam along the rows at −4, −2, 0, 2 and 4 of the MultiPlug. For the beam of 10 × 10 cm^2^: (c) Depth dose curves along the columns at 0, −2 and −4; those at −4 show bigger difference as they are near the beam edge; (d) Cross‐plane profiles. For the beam of 2 × 2 cm: (e) Only the depth dose curve along the column at 0 is plotted; (f) Cross‐plane profiles.

The process commenced by positioning the ArcCHECK‐MR 1, equipped with the MultiPlug, in an iso‐centric manner on the Unity system. Point dose measurements were executed at 25 positions distributed at each ArcCHECK‐MR MultiPlug insert, utilizing a SemiFlex 3D 310021 0.07 cm^3^ ion chamber connected to a UniDos electrometer (both PTW‐Freiburg). The spacing between adjacent measured points was 2.0 cm. These measurements encompassed fields of varying sizes: 2 × 2 cm^2^, 10 × 10 cm^2^, and 40 × 22 cm^2^, respectively. The ensuing depth and cross‐plane profiles, generated from these measurements, were then compared with the corresponding TPS‐calculated data (Figure 2).

**FIGURE 3:**
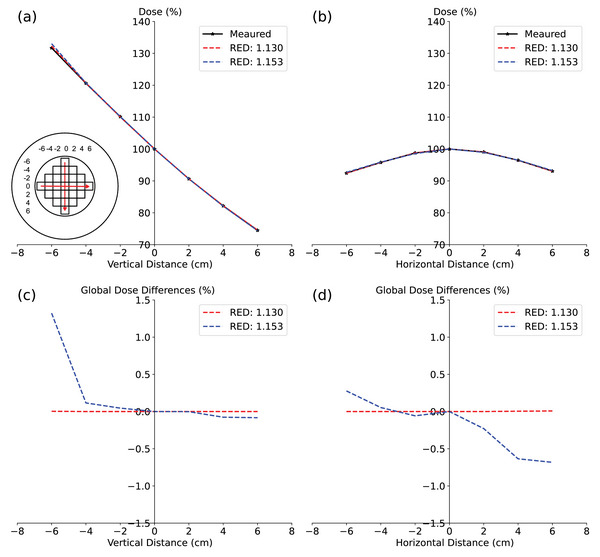
The measured and calculated doses for the MultiPlug in ArcCHECK‐MR 1, using a uniform RED value of 1.130 and 1.153, respectively, were compared to illustrate the dose variations when different RED values are applied. The field size is 40 × 22 *cm* ^2^. This comparison covered: (a) Depth dose curves. (b) Cross‐plane profiles at the isocenter plane. (c) Relative depth dose differences with the two RED values. At the first point (0, −6), the dose difference is 1.32% for RED 1.153 and 0.33% for RED 1.130. (d) Relative cross‐plane dose differences with the two RED values. At the last point (0,6), the dose difference is −0.68% for RED 1.153 and 0.007% for RED 1.130.

To determine the appropriate RED value for PMMA in Monaco, several calculation batches were performed using the GPUMC 7 *FFF* beam model for the Unity 1. A uniform RED was assigned throughout the entire cylindrical volume of the ArcCHECK‐MR phantom, and tested RED values included 1.100, 1.125, 1.130, 1.135, 1.140, and 1.153—the latter based on the specifications of SNC. For each beam in every calculation batch, doses at the 25 central points of the MultiPlug PMMA inserts were sampled using a 0.25 cm radius sphere. The measured and calculated doses for each beam were normalized with respect to the central point doses. Finally, dose discrepancies at sampled points within the beam's jaw opening region were assessed, excluding doses outside the beam aperture from the analysis due to concerns about attenuation by PMMA. Cumulative differences between the measured and calculated doses at each sampled point from all fields were minimized while maintaining consistencies in depth curves and cross‐plane profiles. As a result, the appropriate RED value, 1.130, was confirmed and assigned to components made of PMMA, namely the “Outer,” “Inner,” and “Insert” (Figure 3).

Analysis of the “Detectors” component using MVCT scans revealed high‐density capsules within a contrasting low‐density material. Each capsule contains the active measurement detector, a tiny volume even smaller than individual pixels in the CT image. Adhering to SNC's specifications that establish the SunPoint diode detectors’ resemblance to ion chambers, a reasoned decision led to assigning an RED value of 1.0 to this key component. This value reflects the assumption that the measured signal represents the combined interaction of the active volume, the high‐density capsule, and the surrounding low‐density material.

**FIGURE 4:**
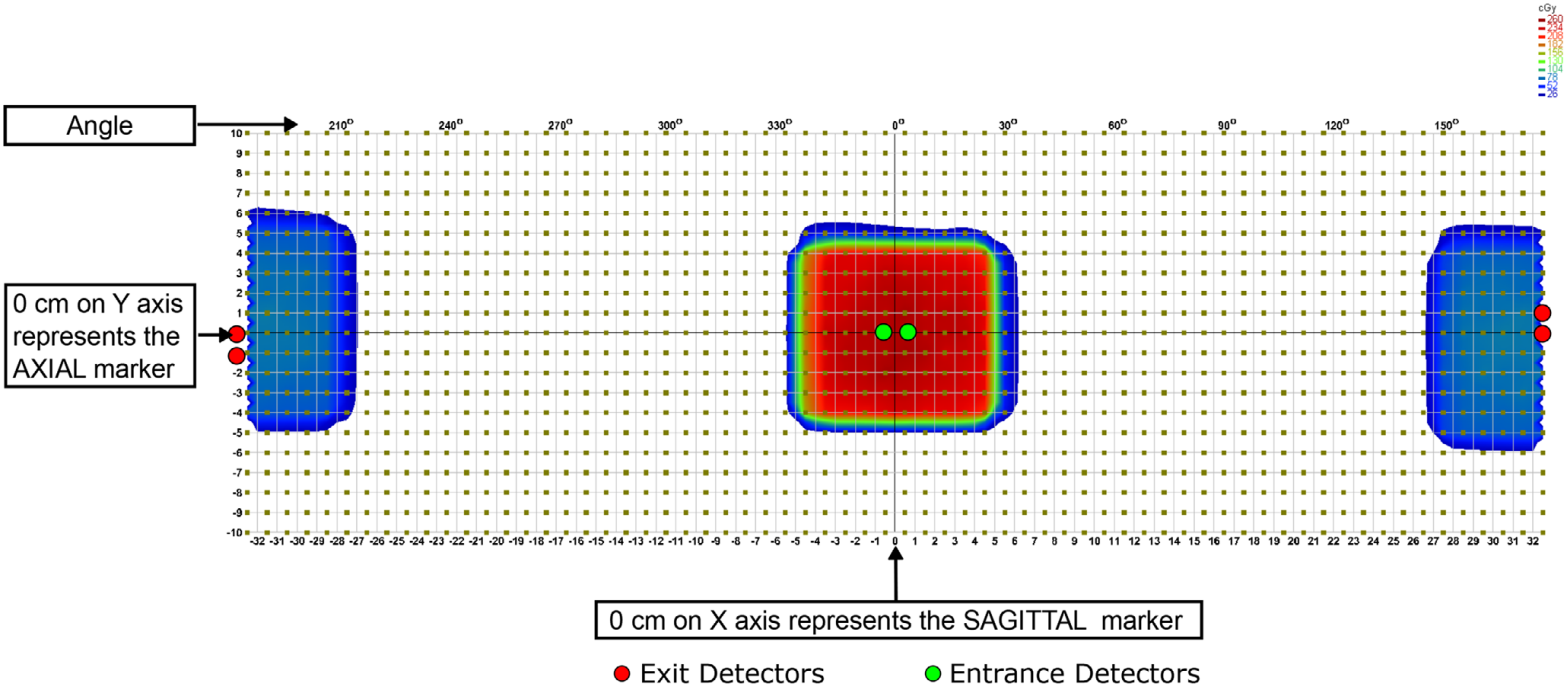
The locations for sampling entrance and exit doses are indicated on the 2D dose map of a 10 × 10 cm^2^ field in SNC Patient software using its proprietary coordinates. There are no detectors located on the beam central axis, for additional information, please consult Figure 5. On the entrance side, one detector is positioned on the left side, and another on the right side. On the exit side, doses are sampled at all four detectors with a 1.0 *cm* pitch. In SNC Patient software dose map chart table, the coordinates of the entrance detectors are (−5, 0) and (5, 0), the coordinates of the exit detectors are (325, 10), (325, 0), (−325, 0) and (−325, −10). Mean doses on the entrance and exit sides are calculated separately, and the EEDR is determined using these mean doses.

To address the “Complex” component, we employed a distinct methodology. The RED value of the “Complex” structure within the virtual phantom was iteratively fine‐tuned in Monaco within the range of 1.130 to 1.250. For each new RED value, the 10 × 10 cm^2^ beam was recalculated, and the updated dose was exported from Monaco. The exported dose was then processed in SNC Patient software, where doses were sampled at the top 2 diodes on both sides of the central axis, for both measured and calculated doses, respectively. The locations where doses were sampled are illustrated in Figure 4.

The calculated and measured mean doses were compared, establishing a ratio. This ratio was used to rescale the Monitor Units (MU) for the 10 × 10 *cm* ^2^ beam in Monaco. Since changing the RED of the “Complex” component affected calculated doses, this adjustment was crucial for absolute dose mode analysis, eliminating the need for further measurements.

Subsequently, the rescaled beam dose was exported and processed within SNC Patient software to calculate local DTA/Gamma passing rates, with a 10% low dose threshold (TH), *γ* (2%/2 mm). The final solution was evaluated based on higher passing rates, while any failed points were meticulously examined.

We employed an alternative approach to calculate the EEDR by sampling doses at specific diode locations within the ArcCHECK‐MR phantom. The top 2 diodes, positioned left and right of the beam central axis, and the bottom 4 diodes surrounding the axis were used, and mean doses were calculated separately for both sets of detectors. The EEDR was determined as the ratio of the mean bottom dose to the mean top dose. This approach aimed to minimize the difference in EEDR between measured and calculated datasets without compromising the *γ* index analysis results. Consequently, we obtained an RED value of 1.200 for the “Complex” structure. Subsequently, we refer to the virtual phantom with RED values as (1.130, 1.200, 1.000, 1.130, 1.130) as the “5‐layer” phantom, and the one with RED values as (1.130, 1.130, 1.130, 1.130, 1.130) as the “RED‐1.130” phantom.

To overcome uncertainties inherent in Monte Carlo statistical fluctuations, we explored an alternative method for setting the calibration dose in ArcCHECK‐MR. This involved calculating a 10 × 10 *cm* ^2^ beam with 200 *MU* on both the “5‐layer” and “RED‐1.130″ phantoms using Monaco”. Instead of directly relying on the point dose from Monaco, we extracted the calculated doses at the positions of the top 2 detectors in the SNC Patient software. This strategy minimizes the impact of uncertainties from Monte Carlo simulations in Monaco and DICOM RT dose unfolding from SNC Patient, ensuring a more reliable representation of the expected calibration dose. These calculated averages were then entered as the “Calibration Dose” field for each phantom within SNC Patient, enabling a direct comparison of measured and calculated doses for each phantom.

The contours of the QA platform from the kV‐CT images were integrated into the 5‐layer phantom. Six distinct objects, labeled as “LtOuter,” “LtMiddle,” “LtInner,” “RtInner,” “RtMiddle,” and “RtOuter,” were assigned their respective uniform RED value. Subsequently, the MR‐linac index couch was imported to complete the contours definition of the virtual phantom and align the virtual phantom properly. Measured data were collected for 10 × 10 cm^2^ beams at gantry angles of 120° and 242°, and the doses of these beams were calculated. Comparisons between the measured data and the corresponding calculated doses for each beam were made in SNC patient software, with the criteria of local DTA/Gamma passing rates, using a 10% low dose TH, and γ (2%/2 mm). The RED values for these structures underwent iterative fine‐tuning within the range of 1.320 to 1.380, until a final solution was achieved, focusing on achieving higher passing rates and meticulously examining any failed points. Ultimately, we determined the appropriate RED value to be 1.350.

### 2.2 Evaluation/validation

#### 2.2.1 Data measured from a conventional linac

Many studies have investigated Unity beam characterization under various scenarios, including both non‐magnetic and 1.5*T* magnetic field conditions, often in water or solid flat phantoms. However, no existing studies have examined the potential influence of the 1.5*T* magnetic field on the calculation of ArcCHECK's EEDR, which could subsequently impact the calculation of RED. Therefore, direct comparisons of dose patterns measured with the ArcCHECK‐MR under both non‐magnetic and 1.5*T* magnetic field conditions would be invaluable for a comprehensive assessment. Due to the unavailability of ArcCHECK‐MR in either institution before the magnet ramped up, data measurements with the ArcCHECK‐MR in a non‐magnetic field condition on the Unity were not feasible. As an alternative, reference data were obtained at a satellite facility of CAMS using an Elekta Axesse linac with a 6 FFF beam. During beam setup, the ArcCHECK‐MR was positioned at an extended source‐to‐central distance (SCD) of 143.5 cm (SSD of 130.2 cm). Jaw openings on the linac were set to 7.0 × 7.0 cm^2^ and 15.4 × 15.4 cm^2^, corresponding to beams of 10 × 10 cm^2^ and 22 × 22 cm^2^ at the center plane of the ArcCHECK‐MR in alignment with the Unity geometry. Measurements of 200 MU were taken for each beam. The measured data from the Axesse linac were used in conjunction with corresponding data from the Unity 2 system to highlight the dose patterns in conditions with and without the 1.5*T* magnetic field. The central helix of the ArcCHECK‐MR, capturing a representative sample of doses across the beam within the full extent of one row of 66 detectors, provided an ideal location to illustrate the agreement between the measured and calculated doses.

#### 2.2.2 Measured and calculated dose patterns at the ArcCHECK‐MR central helix

To assess the accuracy of different phantom models in predicting doses, we conducted an analysis comparing measured and calculated doses in the ArcCHECK‐MR central helix using data acquired on the Unity 2 system. A 22 × 22 cm^2^ beam with a dose of 200 MU was measured at a gantry angle of 0° using the ArcCHECK‐MR 2 on Unity 2. Subsequently, the dose of the same beam was calculated for both the RED‐1.130 and 5‐layer virtual phantoms. The measured and calculated doses were then extracted from the central helix, and differences between the measured and individually calculated data (RED‐1.130 and 5‐layer phantoms) were computed and visualized in a plot.

#### 2.2.3 Effect of QA platform on QA measurements

To investigate the dose attenuation caused by the QA platform, we configured the ArcCHECK‐MR 2 and QA platform in an isocentric setup on the Unity 2. A SemiFlex 3D 300021 ion chamber with a volume of 0.07 cm^3^, connected to a UniDos electrometer, was positioned within the central cavity of the CavityPlug insert in the ArcCHECK‐MR phantom. The output for a 10 × 10 cm^2^ field was measured at every 2° gantry angle, resulting in a total of 174 measurements. Beams in the range of 8° ∼ 18° were excluded due to the hardware protection mechanism of the Unity. The “CCT/couch/QA Platform” dataset was then compared to the TPS calculated dataset in relative dose mode to evaluate the effect of the QA Platform on the QA measurements.

#### 2.2.4 Validating the virtual phantom with unity test plans

The accuracy of virtual phantoms in predicting dosimetry for different beam types was assessed using a set of QA plan templates, following AAPM TG‐119 guidelines.^22^ These templates encompassed simple open fields (10 × 10 cm^2^ beams at gantry angles of 0°, 120°, 180°, and 242°; a 22 × 22 cm^2^ beam at 0°; and specialized APPA and five‐band configurations) and clinical IMRT plans (C‐shape, Head&Neck, Prostate, and MultiTargets). The plans were measured at two collaborating institutions, each utilizing their own site‐specific Unity and ArcCHECK‐MR devices. To address potential device variations, we recalculated the plans using both the 5‐layer virtual phantom and a uniform phantom with adjusted RED values, ensuring a standardized comparison. The agreement between calculated and measured doses was assessed using the DTA and γ index, with different DTA and γ index criteria applied for both local and global evaluations.

## 3 RESULTS

### 3.1 General

#### 3.1.1 The characteristics of ArcCHECK‐MR with CT image analysis

**FIGURE 5:**
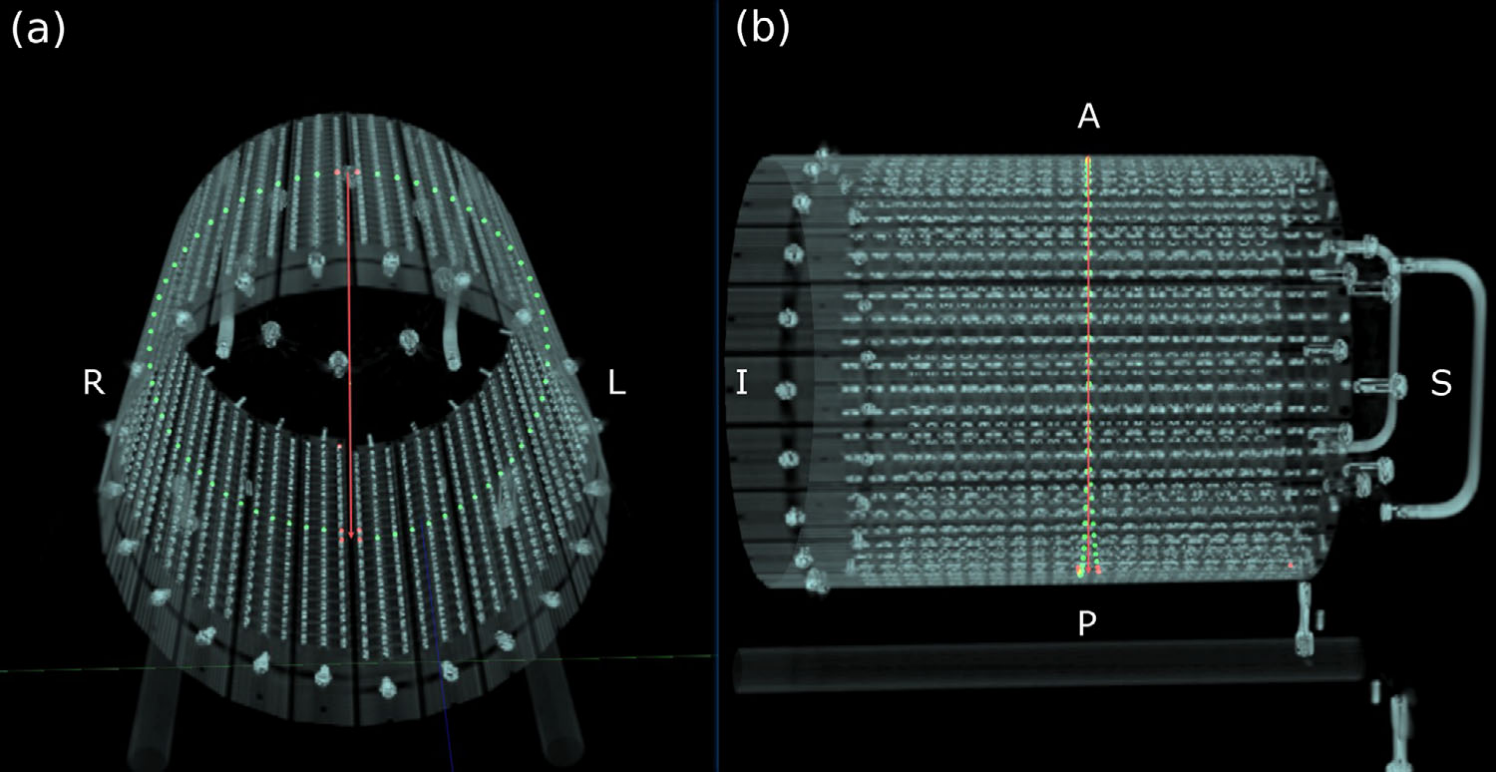
A 3D volume rendering of the ArcCHECK‐MR 2, highlighting its PCBs and detectors. The perspective view (a) and lateral view from the left side (b) show that the device has 22 PCBs, each with 3 detectors in a segment of a helix, totaling 66 detectors in one helix with 1 *cm* spacing between adjacent detectors. The diameter of the detector cylinder array is supposed to be 20.8 *cm*. The detectors on the central helix plus two extra detectors on the exit side, one at the head and another at the tail, are highlighted. The detectors on the entrance and exit sides around the central axis of a beam are displayed in red, while other detectors are shown in green. The red vertical line represents the path of the beam's central axis within the ArcCHECK‐MR. R: right, L: left, A: anterior, P: posterior, S: superior, I: inferior.

Figure 5 shows the 3D rendering of the ArcCHECK‐MR 2 using the dual‐energy KV‐CT images, displayed the high‐density PCBs and detectors within the active measurement volume, encased in high‐density capsules. Notably, no detectors were positioned directly along the central axis of the beam, both on the entrance and exit sides. This was due to the helical alignment of the detectors with a 1.0 cm pitch. As a result, the detectors on the exit side of the central ring were at divergent positions in the DICOM Y direction. This divergence could affect the accuracy of dose when doses on the exit side were sampled directly in Monaco, particularly when precise dose information in this region was required.

**FIGURE 6:**
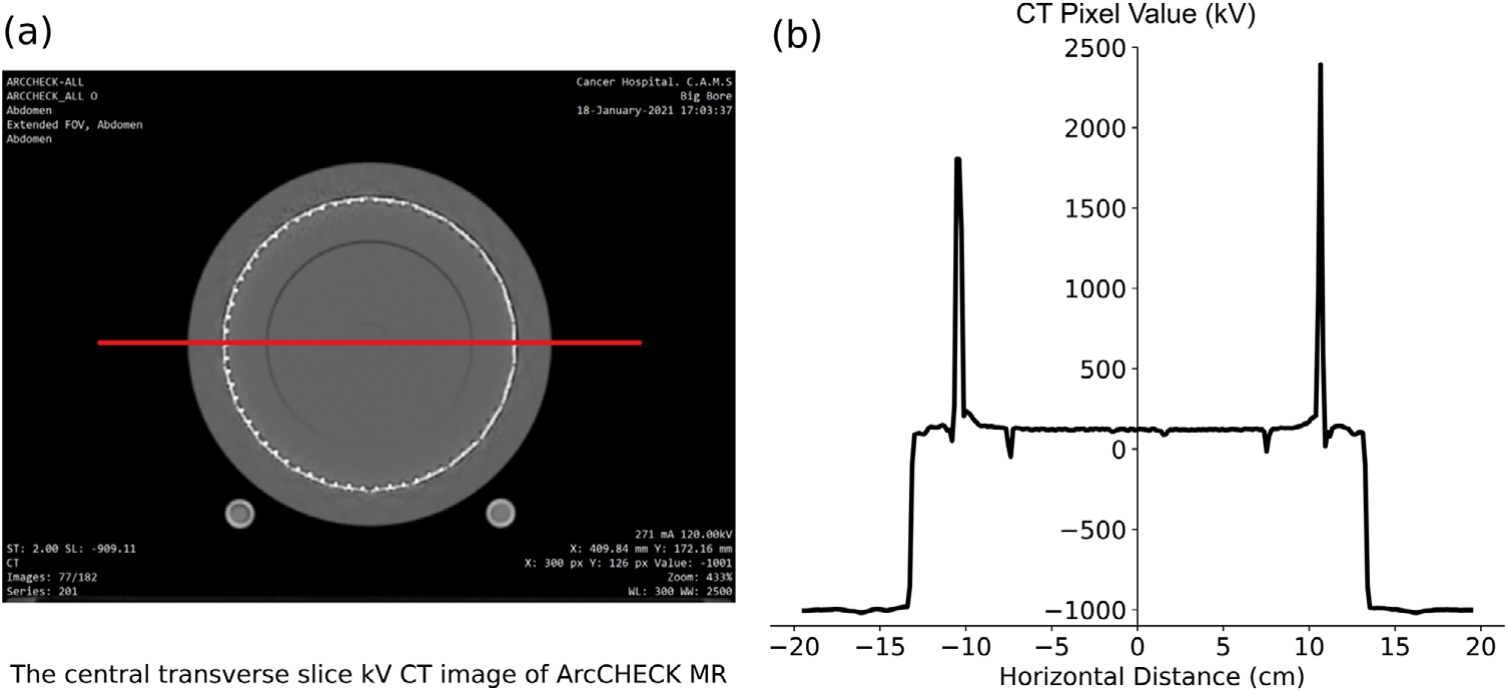
A kV‐CT image of the ArcCHECK‐MR 2 and the corresponding CT pixel values. (a) The central transverse kV‐CT image with a line indicating the location where CT pixel values are sampled. (b) The plot of the sampled kV‐CT pixel values.

Figure 6 shows the CT pixel values plotted along the central horizontal axis of the ArcCHECK‐MR phantom in the kV‐CT image set. It showed variations around the—components, such as the PCBs and detectors, with a maximum value of 2393 at 106.6 mm. Additionally, two distinct notches were observed at ± 7.5 cm on either side of the central axis. These notches coincided with the interface between the inner shell and the CavityPlug insert.

**FIGURE 7:**
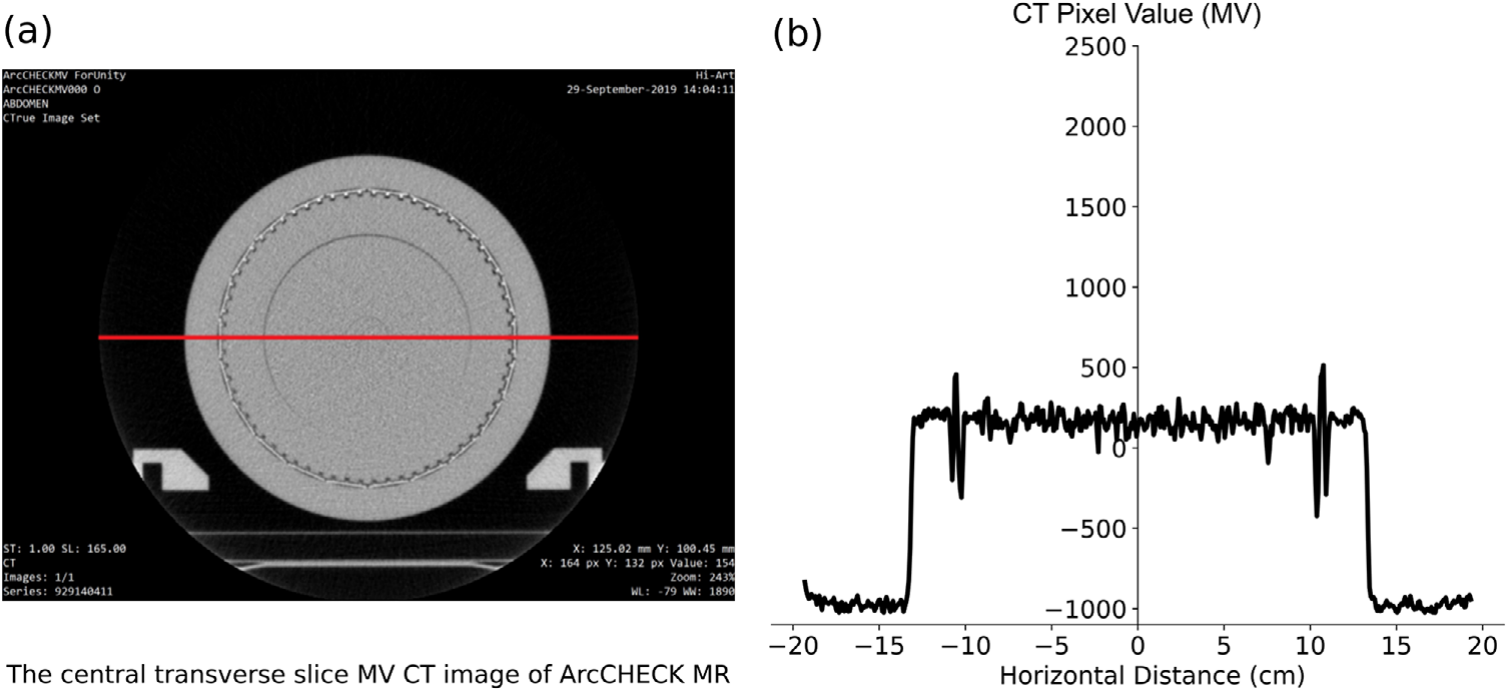
A MV‐CT image of the ArcCHECK‐MR 2 and the corresponding CT pixel values. (a) The central transverse MV‐CT image with a line indicating the location where CT pixel values were sampled. (b) The plot of the sampled MV‐CT pixel values.

Figure 7 shows the CT pixel values plotted along the central horizontal axis of the ArcCHECK‐MR phantom in the MV‐CT dataset. Compared to Figure 6 using kV‐CT, the MV‐CT data revealed similar variations with peaks around the high‐density PCBs and detectors. The maximum pixel value reached 514.5 at 107.6 mm. Only one distinct notch was observed at +7.5 cm, unlike the two notches seen in Figure 6.

Within the range of −10 to 10 *cm* from the isocenter, both the inner shell and insert are composed of PMMA. Figure 6 and 7 illustrate the greater fluctuations in MVCT and kV‐CT pixel values in this region. Statistical analysis is summarized in Table 1, revealing that the standard deviation of MV‐CT pixels is almost double that of kV‐CT (56.5 vs. 27.6). This discrepancy may be attributed to differences in image post‐processing reconstruction methods between kV‐CT and MV‐CT. Despite MV‐CT images displaying fewer metal streak artifacts, the significantly higher uncertainty in MV‐CT pixel values implies potential challenges in accurately representing the PMMA material when MVCT‐to‐RED conversions are utilized in the TPS for treatment planning.

**TABLE 1:** Statistical characterization of CT pixel values extracted from the central image along a defined profile in Figures 6 and 7, respectively.

	Mean	Median	SD^a^	Minimum	Maximum
kV‐CT	124.2	123	27.6	−47.0	234.0
MV‐CT	166.5	168	56.5	−26.5	309.5

*Note*: The data are in the range of −10 *cm* to +10 *cm*, covers the “Inner” and “Insert” PMMA components only. Notably, compared to kV‐CT, MV‐CT data exhibited a larger standard deviation, indicating greater variability in pixel values.

^a^ SD: Standard Deviation.

**FIGURE 8:**
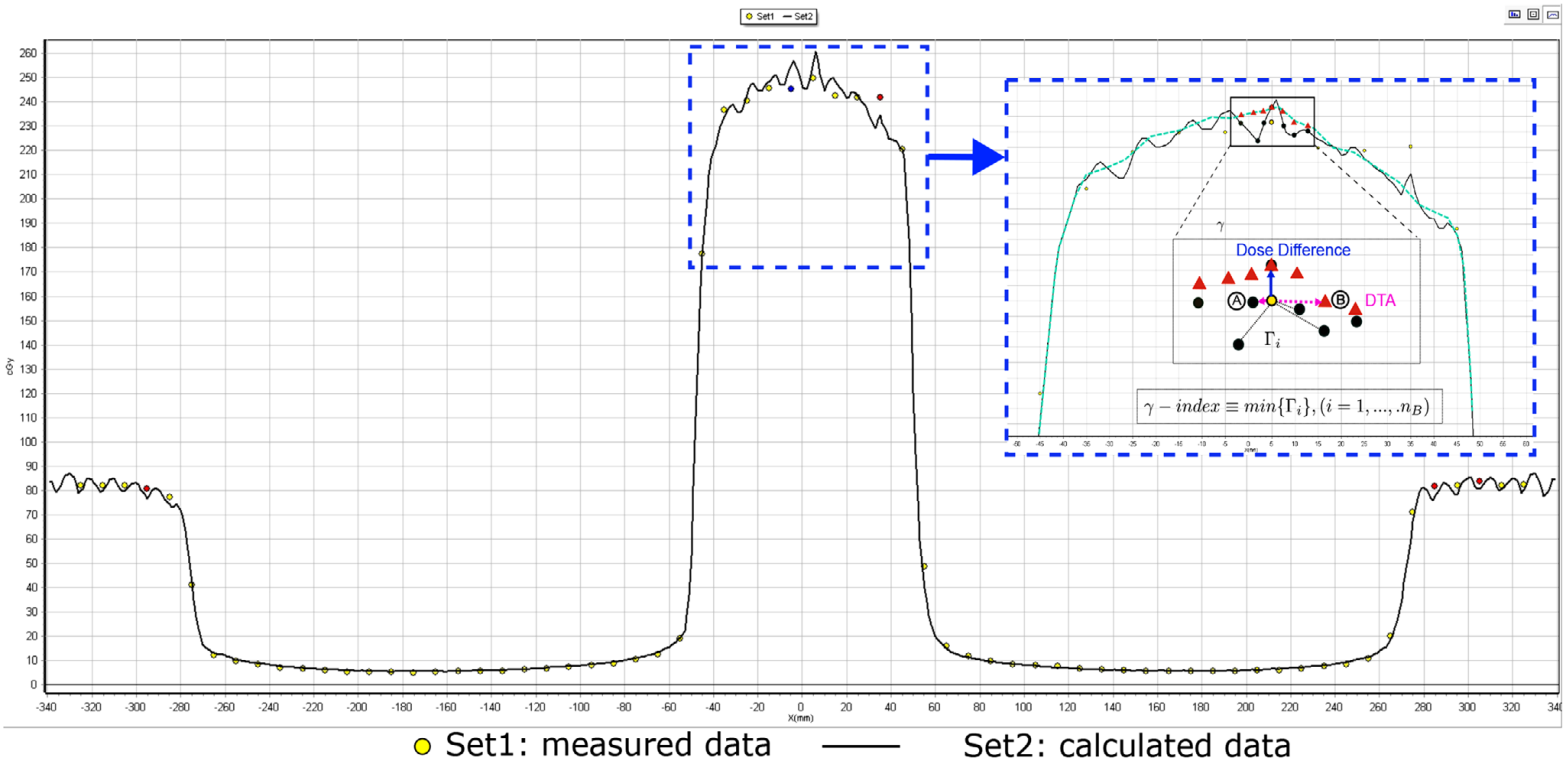
Comparing dose profiles in SNC Patient software for a 10 × 10 cm^2^ beam at gantry 0° reveals noticeable sawtooth patterns in the calculated profile obtained using MVCT‐to‐RED conversion (black line). These patterns introduce inaccuracies in DTA sampling at short distances, potentially misleading the verification of delivered dose against the plan. The inserted image highlights a specific detector near the beam center. The cyan line shows the ideal dose profile for a homogeneous phantom with an overriding RED value of 1.130. DTA sampling points are marked on both profiles: black dots for the MVCT‐to‐RED profile and red triangles for the homogeneous profile. While the absolute dose differences between measured and calculated data are similar, the DTA search algorithm finds a closer point (A) for the MVCT‐to‐RED profile due to the sawtooth pattern, instead of the expected position (B) This can lead to artificially inflated DTA/*γ* index pass rates, potentially masking underlying dose discrepancies and raising concerns about treatment delivery accuracy.

Figure 8 illustrates the comparison between calculated and measured data for a 10 × 10 cm^2^ beam from the ArcCHECK‐MR 2, using MVCT‐to‐RED conversions (without bulk RED overriding) for dose calculation. At the entrance and exit sides, where smooth dose gradients were expected, the calculated profile revealed distinctive sawtooth patterns. These patterns corresponded to changes in CT values between the low‐density wrapping material and the high‐density capsules encasing the detectors. Importantly, these patterns were not caused by statistical uncertainties in the Monte Carlo simulation. The presence of pronounced local dose gradients in unexpected areas could significantly impact dosimetric metrics such as DTA and *γ* index. These metrics rely on comparing dose values at specific points, and the sawtooth patterns might lead to sampling points within local high dose gradients, potentially skewing the results, as depicted in the inserted subpicture of Figure 8.

#### 3.1.2 The ArcCHECK‐MR virtual phantom characteristics

**FIGURE 9:**
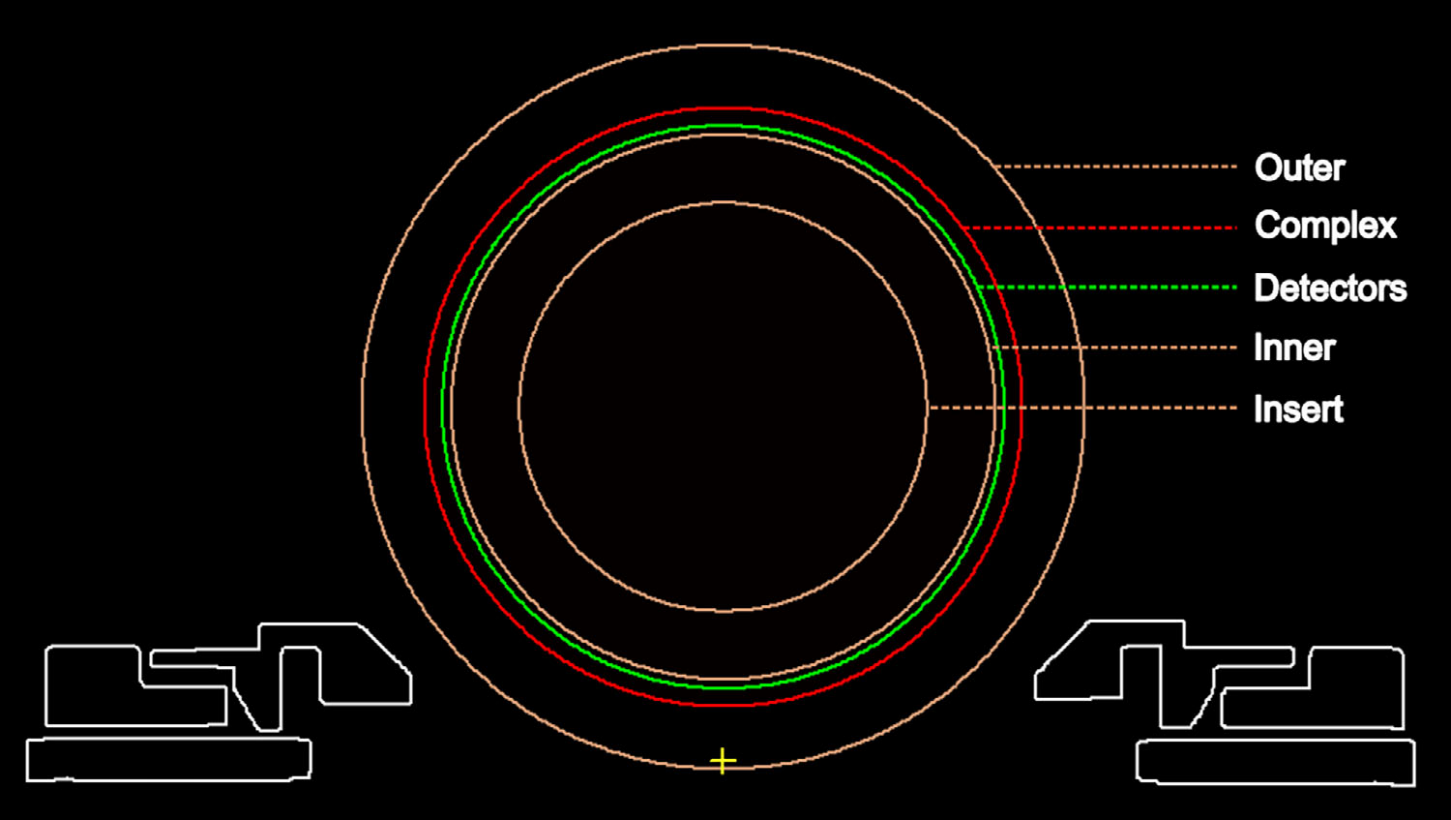
A snapshot of the central transverse slice of the completed 5‐layer phantom. The radii of the five ring structures are as follows: Outer (133.0 mm), Complex (110.0 mm), Detectors (103.4 mm), Inner (100.0 mm), and Insert (75.0 mm). Unlabeled contours represent the QA platform.

Figure 9 presents a transverse view of the five‐layer phantom in Monaco, revealing its structure and composition. We will subsequently explore the key attributes and properties of the ArcCHECK‐MR virtual phantom, illuminating its role in our study and its connection to our research objectives.

### 3.2 Validation

#### 3.2.1 The RED of the PMMA parts

Figure 2 compares measured and calculated dose distributions in relative dose mode, specifically focusing on the MultiPlug inserts for field sizes of 40 × 22 cm^2^, 10 × 10 cm^2^, and 2 × 2 cm^2^ within the RED‐1.130 phantom. For the 40 × 22 cm^2^ field, calculated doses exhibited slight deviations from measured values in the depth dose curves. At points (0 cm, −6 cm) and (0 cm, 6 cm), calculated doses were 0.44% and 0.14% higher, respectively. In the cross‐plane profile, calculated doses were 0.51% higher at the point (0 cm, −6 cm) but −0.94% lower at the point (0 cm, 6 cm).

Figure 3 visualizes the discrepancies between measured and calculated dose distributions within the MultiPlug inserts for a 40 × 22 cm^2^ field, comparing results obtained with RED values of 1.130 and 1.153. Notably, the initial point (a) on the depth dose curve, located at (0 cm, −6 cm), was 7.3 cm from the ArcCHECK‐MR surface. This offset suggests that variations in dose might be even more pronounced at shallower depths, prompting further investigation.

#### 3.2.2 Dose pattern at the ArcCHECK‐MR central helix

**FIGURE 10:**
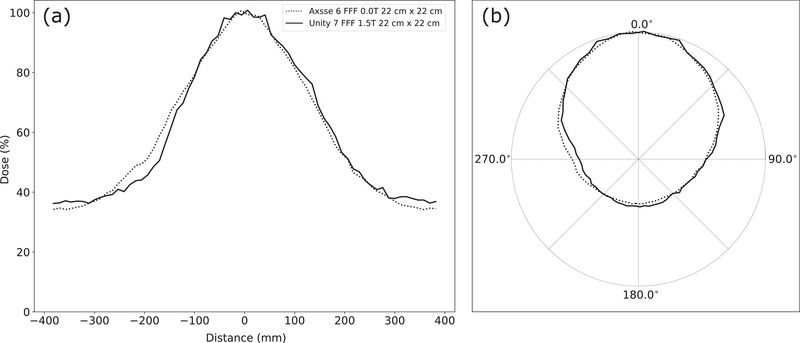
ArcCHECK‐MR central helix dose profiles for nominal 22 × 22 *cm* ^2^ beams. The solid line is the measured data of the Unity 2 7 *FFF* with the ArcCHECK‐MR 2; the dotted line is that of the Axesse 6 *FFF* measured with the ArcCHECK 2. (a) Plots in Cartesian coordinates. (b) Plots in polar coordinates, which accounts for the equal distance of 104 *mm* between each detector and the center of ArcCHECK‐MR. The doses are normalized to the mean dose of the entrance side two detectors for each beam.

Figure 10 illustrates the impact of the 1.5*T* magnetic field on measured dose distributions within the ArcCHECK‐MR 2, focusing specifically on the central helix data for a nominal 22 × 22 *cm* ^2^ field from the 6 *FFF* and 7 *FFF* beams (relative mode). The figure reveals shifts in data points for the Unity 7 *FFF* beam, primarily affecting data points in specific angle ranges, for example, 220° ∼ 315°, and 45° ∼ 90° when the beam is incident at a gantry angle of 0°. It is known that in Unity, the Lorentz force causes the crossline shift.^23^ Note that the Lorentz force direction changes with the secondary electrons’ trajectory, which is relative to the incident photon direction in the 1.5*T* magnetic field. Consequently, the mentioned ranges would vary when the gantry is at different angles. Additionally, on the exit side, Unity data showed higher values in the range of 155° ∼ 205°. This difference was primarily due to the higher PDD values at deeper depths associated with the higher beam quality index of the 7 *FFF* beam (*TPR*_20_ *_/_*_10_: 0.700) compared to the conventional 6 *FFF* beam (*TPR*_20_ *_/_*_10_: 0.680). The changing trajectory of secondary electrons in different densities might also play a role.

#### 3.2.3 Validation the virtual phantom with simple beams

SNC recommended overriding the cylindrical phantom with a uniform RED. We first evaluated the effect of introducing layers with different REDs in the virtual phantom using two simple beams.

**FIGURE 11:**
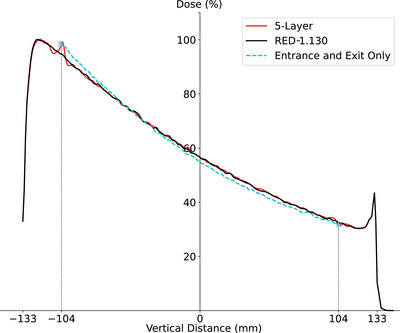
Calculated relative depth dose curves from the RED‐1.130 phantom and the 5‐layer phantom. The calculated doses from the 5‐layer phantom matches the measured entrance and exit doses better than the bulk overridden phantom, RED‐1.130. There are no significant dose differences in PMMA parts. The shorter curve in cyan is a supposed depth dose profile by using measured entrance and exit doses only. Doses are normalized to the dose of the 5‐layer phantom at 13 mm.

Figure 11 shows depth dose curves for a 10 × 10 cm^2^ field. The red and black curves represent calculated data for the RED‐1.130 and 5‐layer virtual phantoms at the beam's central axis. Stars mark measured entrance (−104 mm) and exit (104 mm) doses from the ArcCHECK‐MR 1/Unity 1. The dashed cyan curve illustrated a hypothetical depth dose profile matching these points. The measured EEDR was 0.3207, while the calculated EEDRs were 0.3342 (RED‐1.130) and 0.3229 (5‐layer), resulting in relative differences of 4.2% and 1.0%, respectively. When using the RED‐1.130 phantom dose at −104 mm as the reference in global comparison mode, the 5‐layer phantom exhibited 2.5% higher dose at the same depth, and the exit dose at 104 mm was −0.8% lower in the 5‐layer phantom compared to the RED‐1.130 phantom. Similarly, local comparison showed 2.5% higher and −2.5% lower doses, respectively. A uniform phantom with a different RED value than 1.130 would be needed to match the measured EEDR, affecting scatter dose calculations in the TPS. Introducing two density layers (“Complex” and “Detector”) allowed EEDR matching by adjusting the RED of the “Complex” layer only without changing the RED value of PMMA parts.

**FIGURE 12:**
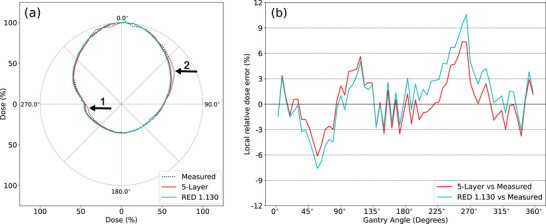
ArcCHECK‐MR central helix dose profiles comparison for a 22 × 22 cm^2^ beam from gantry 0°. (a) The measured data from the ArcCHECK‐MR 2/Unity 2 (dotted in black); the calculated data from the RED‐1.130 phantom (in cyan); and the other calculated from the 5‐layer phantom (in red). Doses are normalized to the mean doses of that being sampled at the two detectors on the entrance side, respectively. The black arrow 1 points to the largest difference at 264° on the left side. The black arrow 2 points to the largest difference at 57° on the right side (b) Local dose differences between the calculated and the measured at each of the detectors.

Figure 12 illustrates comparisons between measured and calculated data for a 22 × 22 cm^2^ field along the central helix. In Figure 12a, the measured data is presented alongside two calculated dose profiles: one using the RED‐1.130 phantom (in cyan) and the other utilizing the 5‐layer phantom (in red). Noticeable trends in dose differences were visible on both sides of the profiles, but the discrepancies between the two calculated profiles were relatively small, with the red profile showing a slight shift closer to the measured data. Figure 12b illustrates local dose differences between calculated and measured data at each detector. In the sector spanning 35° to 80°, the calculated doses were below −3%, with the largest difference occurring at 57°, measuring −7.5% for the RED‐1.130 phantom and −5.8% for the 5‐layer phantom, respectively. Within the sector ranging from 243° to 264° for the RED1.130 phantom and 232° to 298° for the 5‐layer phantom, the calculated doses exceeded 3%, reaching the largest difference of 10.6% at 264° for the RED‐1.130 phantom and 7.3% for the 5‐layer phantom. Note that detectors in the sectors of 35° to 80° and 35° to 80° were irradiated laterally in this scenario, subjecting them to the effect of angular dependency. For clinical IMRT treatments, where the aperture size of beamlets is much smaller, lateral incidents are rare unless the target volume is located far away from the central axis.

#### 3.2.4 Effect of QA platform on QA measurements

**FIGURE 13:**
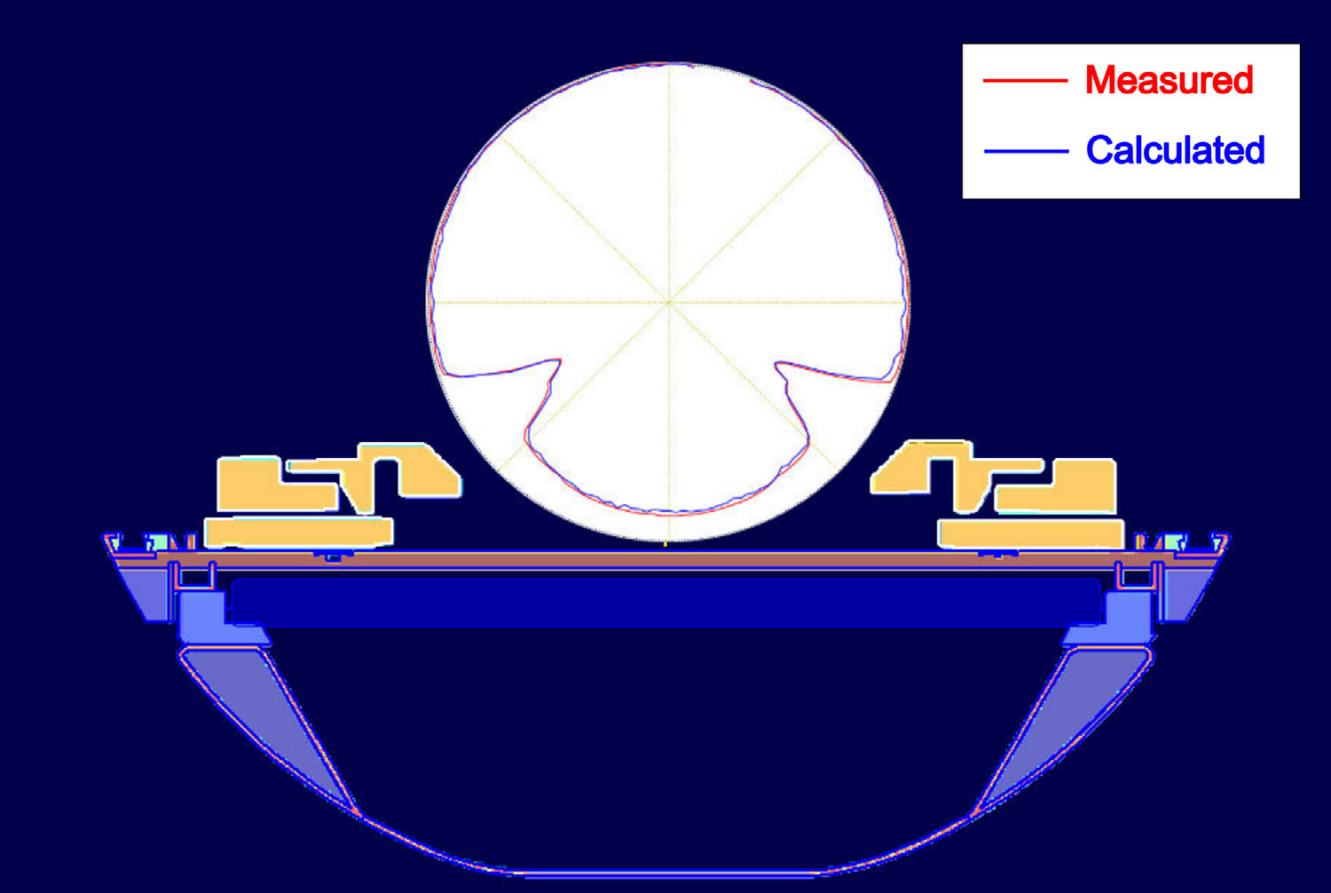
Variations in central axis dose attenuation were observed at different gantry angles in the presence of the MR cryostat, couch, post coil, and QA Platform during patient‐specific quality assurance tests using ArcCHECK‐MR. The dataset comprises data from 174 beams, each with a field size of 10 × 10 *cm* ^2^ at the isocenter, and gantry angles ranging from 180° to 178° in 2° increments, excluding beams within the range of 8° to 18°, 100 *MU* were delivered for each beam. The red curve represents the measured data, while the blue curve corresponds to the calculated data. A SemiFlex 3D 300021 0.07 *cm* ^3^ ion chamber connected to a UniDos Electrometer was placed inside the central cavity of the ArcCHECK‐MR phantom to measure data. Doses are normalized to the doses at the isocenter of the beam at the gantry 0°, respectively. Note that the introduction of the QA Platform results in additional attenuations not typically encountered in clinical treatment modes. The background image provides a REDs layer view of the various components, including the couch, post coil, QA Platform, as well as the ArcCHECK‐MR. For visualizing dose profiles, the RED of the ArcCHECK‐MR is forced as 1.130, but not the values of different layers of the 5‐layer phantom. The attenuations at different angles due to ArcCHECK‐MR itself are assumed to be uniform.

Figure 13 illustrates a comparison between the measured and calculated attenuation patterns obtained from the ArcCHECK‐MR 2/Unity 2 for a 10 × 10 cm^2^ field. Data were collected at every 2° gantry step during a full rotation of the Unity gantry, excluding beams in the range of 8° ∼ 18°. The presence of the couch and post coil resulted in an 8% reduction in gantry angles between 135° and 225°. Furthermore, when using the ArcCHECK‐MR with the QA platform, higher attenuation levels were observed in the angle ranges of 110° to 135° and 225° to 250°. The outputs at angles of 120° and 242° were approximately 0.5.

#### 3.2.5 Validation the virtual phantom with the unity test plans

The test plans for Unity were computed based on the parameters specified in the treatment planning section, utilizing a 5‐layer virtual phantom. Following SNC's guidelines, these test beams were additionally calculated using a phantom with a RED override method. Specifically, an RED value of 1.130 was applied for ArcCHECK‐MR 1. However, due to ArcCHECK‐MR 2 having a higher EEDR, an RED value of 1.170 was used as an override to match the EEDR.

Two sets of criteria were utilized in SNC Patient software analysis. Set 1 consisted of a TH of 10%, γ (2%/2 mm), and set 2 consisted of a TH of 10%, γ (3%/2 mm). The results from both the uniform phantom and the 5‐layer phantom were compared and presented. The results for the ArcCHECK‐MR 1/Unity 1 are summarized in Table 2, and those for the ArcCHECK‐MR 2/Unity 2 are summarized in Table 3.

**TABLE 2:** Unity test beams passing rates summary for the ArcCHECK‐MR 1/Unity 1.^a^

	2%/2 mm								3%/2 mm							
	Uniform REDs^b^				5‐layer REDs^c^				Uniform REDs				5‐layer REDs			
	Local		Global		Local		Global		Local		Global		Local		Global	
Plan ID	DTA	Gamma	DTA	Gamma	DTA	Gamma	DTA	Gamma	DTA	Gamma	DTA	Gamma	DTA	Gamma	DTA	Gamma
10 × 10	62.9	63.7	97.7	97.7	87.9	88.3	96.1	95.7	75.0	74.2	100.0	100.0	96.1	95.7	99.2	98.8
G180	75.6	79.3	91.9	93.6	88.2	88.6	93.5	93.9	89.8	90.7	97.2	97.6	93.8	96.7	98.8	100.0
G242	76.1	77.8	93.8	94.2	81.6	84.1	94.7	95.1	83.1	80.0	96.3	96.3	85.7	89.4	96.3	96.7
G120	72.0	76.4	96.5	98.8	84.3	87.8	93.7	96.9	81.9	84.6	99.2	99.6	89.4	91.7	98.4	99.2
APPA	96.0	95.7	97.8	99.3	94.6	95.0	96.8	98.2	98.2	98.2	100.0	100.0	97.1	96.8	98.9	100.0
22 × 22	36.4	37.7	78.6	79.0	65.2	66.3	92.1	92.4	49.8	49.6	91.3	91.6	78.6	78.4	98.7	98.7
5Bands	91.7	90.0	97.8	97.5	89.3	86.9	97.5	97.5	95.8	95.0	98.2	99.4	92.3	91.5	99.0	99.6
Cshape	85.7	85.5	93.8	95.8	78.4	79.5	89.8	93.5	87.1	88.5	96.1	97.9	82.1	83.8	94.2	97.0
Head&Neck	83.3	85.9	99.5	100.0	77.9	80.6	98.6	99.7	87.0	90.8	99.9	100.0	83.1	88.0	100.0	100.0
Prostate	81.9	83.8	92.7	94.3	76.3	79.9	91.9	94.1	83.0	85.6	94.5	97.0	78.5	83.2	94.9	95.9
MultiTargets	46.2	45.8	75.1	80.2	41.4	41.1	68.5	75.0	47.8	49.6	85.6	88.4	43.1	44.5	83.3	86.9

^a^ Data were measured when the “Out Of Beam Corrections” of “Measurement” preference in SNC Patient Software was not checked.

^b^ Uniform REDs: 1.130, 1.130, 1.130, 1.130, 1.130.

^c^ 5‐layer REDs: 1.130, 1.200, 1.000, 1.130, 1.130.

**TABLE 3:** Unity test beams passing rates summary for the ArcCHECK‐MR 2/Unity 2.^a^

	2%/2 mm								3%/2 mm							
	Uniform REDs^b^				5‐layer REDs^c^				Uniform REDs				5‐layer REDs			
	Local		Global		Local		Global		Local		Global		Local		Global	
Plan ID	DTA	Gamma	DTA	Gamma	DTA	Gamma	DTA	Gamma	DTA	Gamma	DTA	Gamma	DTA	Gamma	DTA	Gamma
10 × 10	73.0	73.8	98.4	98.8	88.3	87.5	94.9	95.7	75.0	86.7	85.9	100.0	100.0	93.8	98.0	98.0
G180	81.0	81.8	93.4	93.4	87.6	88.8	93.8	94.6	89.8	89.3	88.8	95.0	96.3	93.4	95.5	97.1
G242	80.4	84.1	96.7	97.1	93.1	93.5	98.8	98.8	83.1	88.2	89.8	97.1	97.6	98.0	99.6	100.0
G120	70.0	73.5	92.1	94.5	74.2	76.3	87.7	90.5	81.9	77.5	78.3	85.3	96.8	82.1	92.1	94.4
APPA	96.0	96.0	96.4	96.4	93.8	93.5	94.6	94.9	98.2	97.5	97.1	97.5	99.3	96.0	97.1	98.9
22 × 22	84.9	84.9	98.0	98.6	84.6	85.7	98.0	98.9	49.8	87.3	88.1	99.6	100.0	88.2	100.0	100.0
5Bands	93.0	93.0	97.1	98.3	93.6	93.0	97.3	98.3	95.8	94.2	95.1	98.4	98.9	95.2	99.1	99.2
Cshape	85.8	87.7	97.8	98.6	85.9	86.6	97.7	98.9	87.1	88.4	91.5	99.3	99.7	91.4	99.2	99.7
Head&Neck	83.3	84.1	93.8	95.9	83.1	83.5	93.8	96.7	87.0	84.7	87.6	95.9	98.1	88.0	95.5	98.6
Prostate	57.9	59.3	78.0	79.9	59.8	61.4	79.9	80.7	83.0	60.1	63.1	84.7	85.7	64.2	85.2	86.3
MultiTargets	41.3	42.4	77.1	77.3	57.3	58.1	82.8	81.7	47.8	55.1	54.6	87.7	88.1	70.8	93.7	92.6

^a^ Data were measured when the “Out Of Beam Corrections” of “Measurement” preference in SNC Patient Software was checked.

^b^ Uniform REDs: 1.170, 1.170, 1.170, 1.170, 1.170. (The value of 1.170 was used for matching the ArcCHECK‐MR specific EEDR.).

^c^ 5‐layer REDs: 1.130, 1.200, 1.000, 1.130, 1.130.

We compared the performance of a 5‐layer phantom with a conventional uniform RED1.130 phantom. The 5‐layer phantom exhibited higher passing rates when using global γ criteria of γ (2%/2 mm) and γ (3%/2 mm). Specifically, the local *γ* index for open beams with the 5‐layer phantom was 5∼7% higher than that with the RED‐1.130 phantom, and for segmented IMRT beams, it was 2∼4% higher. Notably, the passing rates for the “Prostate” and “MultiTargets” plans were lower for both phantoms, but the underlying reasons were not investigated in this study.

Aligning the virtual phantom with the isocenter streamlined the process of accepting the default (0, 0, 0) isocenter coordinates when opening the calculated QA plan DICOM RT Dose file in SNC Patient software, resulting in significant time savings.

## 4 DISCUSSION

The primary goal of a patient‐specific plan QA device is to accurately compare the calculated dose with the measured absolute dose from a dosimeter, ensuring minimal dosimeterrelated uncertainties to enhance error detection during the planning and delivery stages. In this study, we propose a solution that involves integrating a standardized 3D volumetric virtual phantom for patient‐specific QA into the MRgRT TPS.

Our analysis of both kV‐CT and MV‐CT images revealed that the ArcCHECK‐MR phantom comprises a variety of densities, including high‐density materials like PCBs and capsules, as well as low‐density regions. In our study, we specifically characterized the RED for PMMA by comparing calculated doses with measured data at 25 points inside the MultiPlug of the ArcCHECK‐MR. This characterization enabled us to assign an RED value of 1.130 in Monaco for PMMA components, including the “Outer,” “Inner,” and “Insert” of the ArcCHECK‐MR. Subsequently, we focused on simulating the change in the trajectory of secondary electrons in the TPS dose calculation process for regions containing PCBs and the detector array. We aimed to match the EEDR commonly associated with the ArcCHECK‐MR. Assuming that the RED of the effective measurement volume of the detector arrays could be approximated as 1.000, adjustments were made only to the “complex” component.

Many physicists’ immediate response upon encountering inhomogeneous materials within a QA phantom is to conduct MVCT scans, followed by applying MVCT‐to‐RED conversions to gather RED information and assess the impact of inhomogeneity. However, the QA tool itself has already compensated for the effects of inhomogeneity through calibration.^24^ Simultaneously, as illustrated in Figure 8, corrections based on MVCT‐to‐RED conversions can introduce local changes in dose gradients in calculated doses, particularly in areas where we expect small dose gradients. The algorithm is more likely to identify sampling points meeting DTA criteria in the calculated dose close to the evaluation point, thereby reducing DTA and the corresponding gamma index values and resulting in a higher pass rate. Therefore, using an ArcCHECK‐MR phantom based on MVCT scan images in clinical QA should be avoided.

van Zijp et al. reported high electron‐density materials could significantly impact the size of the dose kernel, influencing the magnetic field's effect on dose deposition reported that accurate geometrical QA tests in a 1.5*T* magnetic field, and 2 ∼ 3 mm thick copper plates is required to reduce the electron path length.^15^ However, the high‐density capsule of the SunPoint detector might not be thick enough to achieve this range. McDonald et al.^12^ demonstrated that hot and cold spots in the dose distribution occur at interfaces between high‐ and low‐density regions. Our approach resulted in similar changes in depth dose curves due to the ERE induced by the 1.5*T* magnetic field, while also ensuring that local high dose gradient regions in the lateral dose profiles, used by SNC Patient software for DTA/gamma analysis, were avoided.

Reynolds^13^ emphasized the significance of the relative orientations of the photon beam, magnetic field, and radiation detector in influencing the dose response of radiation detectors. The presence of high‐density encapsulation can also modify the electron trajectories under the influence of the magnetic field. Houweling et al.^19^ observed slightly higher standard deviations in inter‐diode dose responses for an MR‐linac compared to a conventional linac. In Figure 10, we present our analysis of 22 × 22 cm^2^ beam data obtained from both a conventional linear accelerator with 6 *FFF* beams and Unity with 7 *FFF* beams. Notably, we have identified distinct patterns in the data points, particularly on the range of 220° ∼ 315°, as well as that of 45° ∼ 90°. As Woodings et al. reported,^23^ the Lorentz force causes a crossline shift in water. The presence of interfaces between materials of varying densities within the ArcCHECK‐MR might amplify this effect. While the TPS can account for this shift in homogenous media like water and for heterogeneities in the human body, further research is needed to investigate how to accurately account for such significant density variations in patient QA devices like the ArcCHECK‐MR. This will improve dosimetric accuracy in patient QA and ensure optimal treatment delivery.

As noted in the literature by Houweling et al.,^19^ SNC Patient software incorporates a correction factor that depends on factors like beam profile and, importantly, energy. When saving a measurement file, the software requests information about the linac vendor and beam energy. However, in version 8.3, there is no explicit option for 7 *MV* beams. Users can choose between 6 *MV*, 6 *FFF* or 8 *MV*, potentially leading to the application of different correction factors to the measured signals, thus affecting the RED determination accuracy. Our analysis of the EEDR using Monaco revealed a consistent ratio of 0.3264 when assuming an energy of 8*MV* and assigning a RED of 1.130 to all PMMA components (Outer, Inner, and Insert). However, for other energy options, adjustments to the RED of the Insert were necessary to match the calculated and measured ratios. SNC has confirmed that the newer version of Patient software (version 8.5) includes an energy option as 7 *FFF* beams. The introduction of this new option requires evaluation to determine its impact on EEDR calculations and RED determination accuracy.

In this study, we proposed a new method of calculating the EEDR by considering the helical alignment of diodes. The measured results from two systems showed differences of up to 2%. While we assumed identical hardware geometries for the ArcCHECK‐MR units used in this study, it's important to acknowledge the potential for variations between units, particularly in the detector array calibration factors provided by SNC. To match the EEDR of each unit, we employed RED values of 1.130 for the ArcCHECK‐MR 1 and 1.170 for the ArcCHECK‐MR 2 in the RED override calculation. Recently we learned from other two sites that their measured EEDR values were 0.3345 and 0.3120, respectively. (Private communication, unpublished data). If EEDR variations are a concern, adjustments can be made within the 5‐layer virtual phantom by modifying the “Complex” structure's RED while keeping other parameters constant. This approach is straightforward since the PMMA characteristics should remain consistent from the same vendor. Consequently, the resulting RED values from different ArcCHECK‐MR units can serve as a tool to evaluate the long‐term consistency of the vendor's detector array calibration factors or PMMA characteristics, both within a single institution and across multiple institutes’ studies.

The QA platform serves as an adapter for various QA devices, ensuring precise positioning within the Unity bore. However, it possesses a considerable size and weight and may not fit entirely within the field of view of a large bore CT scanner. To address this issue, we have proposed a straightforward solution by incorporating the QA platform into the virtual phantom as multiple contours. Recently, Subashi et al. ^21^ observed a decline in QA pass rates within specific gantry angle sectors, attributing it to couch‐related attenuation of high‐density materials. In our study, we have substantiated that most attenuation within these gantry sectors, namely 110° ∼ 135° and 225° ∼ 250°, is primarily caused by the QA platform. It's essential to note that the QA platform is absent during actual treatment when the beam occupies these gantry sectors. This distinction should be kept in mind when interpreting QA outcomes. Our analysis revealed that in the test plans employed for this study, the “Prostate” and “Multitarget” plans exhibited lower pass rates. Both plans featured beams at gantry angles of 120° and 240°. It is advisable to review and modify these plans to reduce this artifact from the QA platform. There is a growing demand for a lighter, simpler support cradle that can be accurately positioned with minimal radiation attenuation.

Our findings, particularly when applying the more rigorous γ (2%/2 mm) criteria in local mode, indicate higher pass rates and underscore the potential value of the virtual phantom. However, no improvements are observed with the γ (2%/2 mm) criteria in global mode. Moreover, the pass rates for clinical plans decrease further when employing γ (3%/2 mm) criteria in both local and global modes. As emphasized in TG‐218 by Miften et al.,^25^ that the γ test may underestimate the clinical implications of specific dose delivery errors. We also acknowledge the sensitivity concerns raised by Kry et al.,^26^ regarding measurement based IMRT QA methods. It may be beneficial to explore more test plans with known reasons for dose delivery errors to justify the use of stringent criteria with ArcCHECK‐MR.

To measure a set of non‐magnetic field beam data with ArcCHECK‐MR requires careful consideration and preparation in advance. The study was conducted at the early stage when Unity was cleared for clinical use, it was unfeasible to acquire non‐magnetic beam data with the ArcCHECK‐MR since the ArcCHECK‐MR was not available before the magnet was ramped up. As an alternative, we utilized data from a 6 *FFF* beam produced by a conventional linear accelerator as a reference for non‐magnetic conditions, despite its different beam quality index compared to a 7 *FFF* beam. In future work, it may be beneficial to explore the feasibility of inserting a cerrobend block of appropriate thickness into the path of the 6 *FFF* beam on a conventional linac to better match the beam quality index and simulate the conditions of a 7 *FFF* beam in a non‐magnetic field.

The ion chamber SemiFlex 31021 0.07 cm^3^ detector was used to measure multiple points doses within the insert to calculate the PMMA RED. It's worth mentioning that a SunPoint detector, which is identical to the detectors integrated into the ArcCHECK‐MR system, may provide a more accurate simulation of the PMMA and diode detector interaction within the 1.5*T* magnetic field.

This virtual phantom can be adopted by other institutions through the addition of the phantom data files directly to the TPS specified working directory. Subsequently, the RED value of the “Complex” layer should be adjusted based on their site‐specific EEDR measurements from the ArcCHECK‐MR. The integration of the virtual ArcCHECK‐MR phantom with readily available beam and plan templates, adhering to AAPM‐TG 119, has the potential to significantly improve dosimetry comparisons across multiple institutions.

## 5 CONCLUSIONS

In this study, we built a virtual phantom for ArcCHECK‐MR and integrated it into a TPS. The RED values of our phantoms allowed us to simulate the ERE resulting from transitions between different density areas within the real phantom while exposed to the 1.5*T* magnetic field of Unity. We determined the RED of PMMA that made of the ArcCHECK‐MR through independent measurements. Additionally, we characterized the REDs of both high‐density and lower‐density regions to ensure consistent results across various MR‐linac and ArcCHECK‐MR units. This approach not only reduces uncertainty in the creation of QA phantoms at individual institutions but also enhances our understanding of the QA device's capabilities and be able to evaluate the consistency of its array calibration factors when more data are available.

## AUTHOR CONTRIBUTIONS

MJ designed the study, interpreted data, performed statistical analysis and drafted the manuscript. ZL, YT, KZ and ML collected and interpreted data. YC contributed significantly to the discussion and interpretation of the results. All co‐authors read and revised the manuscript. The final version of the manuscript was approved by all co‐authors.

## CONFLICT OF INTEREST STATEMENT

The authors declare no conflicts of interest.
